# Editorial: Recent advances in sleep and circadian rhythms: the hypothalamus and its relationship with appetite

**DOI:** 10.3389/fnins.2024.1385619

**Published:** 2024-02-29

**Authors:** Henrik Oster, Christopher S. Colwell

**Affiliations:** ^1^Center of Brain, Behavior and Metabolism, Institute of Neurobiology, University of Lübeck, Lübeck, Germany; ^2^UCLA Laboratory of Sleep and Circadian Medicine, Psychiatry and Biobehavioral Sciences, University of California, Los Angeles, Los Angeles, CA, United States

**Keywords:** sleep, hypothalamus, circadian rhythm, appetite, energy metabolism

The circadian clock is a distributed cellular timing system that coordinates physiological processes across the 24-h day cycle. At the molecular level, a handful of ubiquitously expressed clock genes and proteins are organized in an interlocked system of feedback loops that coordinate rhythmic expression of physiologically active target genes in a tissue-specific manner. It is estimated that about 5–10% of all active genes in each tissue are under circadian control (Hughes et al., 2017). A circadian pacemaker in the hypothalamic suprachiasmatic nucleus (SCN) coordinates cellular clocks across the body with each other and with external time (Colwell, 2011; Pilorz et al., 2018). Such clocks have been described in many peripheral tissues, but also in different regions of the hypothalamus (Guilding et al., 2009). Here, they have been implicated in appetite regulation across the day and energy homeostasis (Cedernaes et al., 2019a; Sayar-Atasoy et al., 2024).

Major outputs of the circadian clock are sleep-wake behavior and energy metabolism (Koop and Oster, 2022). The SCN pacemaker directly or indirectly controls activity of sleep- and arousal- regulating neurons of the basal forebrain (galaninergic neurons of the ventrolateral preoptic area), the lateral hypothalamus (orexin/hypocretin neurons), and the brainstem (noradrenergic neurons of the ascending arousal system). Temporal information from the SCN reaches these circuits through indirect innervation (e.g., through the dorsomedial nucleus of the hypothalamus) or neuroendocrine signals such as pineal melatonin (Saper et al., 2005; Saper and Fuller, 2017).

Circadian control of energy metabolism is achieved through rhythmic hormone release (e.g., of cortisol from the adrenal or insulin from pancreatic beta cells), coordination of metabolic tissue circadian clocks, and through the regulation of appetite and energy expenditure regulatory circuits in the basal hypothalamus (Cedernaes et al., 2019b). Sleep and food intake are mutually exclusive processes, and circadian circuits in the mediobasal and lateral hypothalamus coordinate this interaction and help maintaining metabolic homeostasis.

In this Research Topic, we highlight recent advances in studying the interaction between sleep, hypothalamic circadian rhythms, and the regulation of appetite and energy intake. Bouâouda and Jha summarize what we know about the role of hypothalamic melanocortin-orexin crosstalk as an important integrator of daily appetite and sleep rhythms. Orexin-deficient patients suffer from narcolepsy and high sleep pressure while at the same time showing deregulated appetite signaling with increased food intake and craving for high-density food items (Mogavero et al., 2023). In line with this, anti-narcoleptic therapies such as sodium oxybate (SXB) often come with metabolic side effects such as appetite suppression (Lecendreux et al., 2022). The role of circadian clocks or even the hypothalamus in these drug effects remain unclear.

Disruption of regular sleep-wake rhythms, sleep curtailment, or mistimed sleep have been shown to affect appetite, food choice and energy expenditure—all of which are under control by hypothalamic circuits and neuroendocrine outputs (Chamorro et al., 2023). At the same time, this region of the brain is highly sensitive to metabolic feedback signals from the periphery in the form of hormones or metabolites (Friedman, 2019; Mitchell and Begg, 2021). These can act directly on the activity of orexigenic and anorexigenic nuclei or through resetting molecular circadian clocks in these areas (Tsang et al., 2020), thus modulating activity through circadian transcriptional programs. Such meal-related circadian adaptation is termed *food entrainment*, and two studies in this Research Topic describe mediators of this process—oxytocin and leptin (Caba et al.; Sun et al.).

In summary, there is a growing body of work indicating that the hypothalamus plays a critical role in integrating sleep, circadian rhythms, and metabolism. The hypothalamic centers regulating these processes are closely intertwined and can be difficult or even impossible to disentangle. A better understanding of these interactions will help in devising therapeutic strategies to some of the most prevalent disorders including Type-2 diabetes, obesity, and sleep disruption.

## Author contributions

HO: Writing – original draft, Writing – review & editing. CC: Writing – original draft, Writing – review & editing.
